# The Correlation of Hemostatic Parameters with the Development of Early Sepsis-Associated Encephalopathy. A Retrospective Observational Study

**DOI:** 10.2478/jccm-2024-0040

**Published:** 2024-10-31

**Authors:** Florin Scarlatescu, Ecaterina Scarlatescu, Dana Rodica Tomescu, Daniela Bartos

**Affiliations:** Clinical Emergency Hospital Bucharest, Romania; Carol Davila University of Medicine and Pharmacy, Bucharest, Romania; Fundeni Clinical Institute, Bucharest, Romania

**Keywords:** sepsis-associated encephalopathy, platelets, rotation thromboelastometry, clotting, clot strength

## Abstract

**Introduction:**

Sepsis-associated encephalopathy (SAE) is one of the most common complications seen both in early and late stages of sepsis, with a wide spectrum of clinical manifestations ranging from mild neurological dysfunction to delirium and coma. The pathophysiology of SAE is still not completely understood, and the diagnosis can be challenging especially in early stages of sepsis and in patients with subtle symptoms.

**Aim of the study:**

The objective of this study was to assess the coagulation profile in patients with early SAE and to compare the hemostatic parameters between septic patients with and without SAE in the first 24 hours from sepsis diagnosis.

**Material and methods:**

This retrospective observational study included 280 patients with sepsis in the first 24 hours after sepsis diagnosis. A complete blood count was available in all patients; a complex hemostatic assessment including standard coagulation tests, plasmatic levels of coagulation factors, inhibitors, D-dimers, and Rotation thromboelastometry (ROTEM, Instrumentation Laboratory) was performed in a subgroup of patients.

**Results:**

Early SAE was diagnosed in 184 patients (65.7%) and was correlated with a higher platelet count, after adjusting for age and leucocyte count. Compared to patients without neurological dysfunction, patients with early SAE presented a more active coagulation system revealed by faster propagation phase, increased clot firmness and elasticity with a higher platelet contribution to clot strength. The initiation of coagulation and clot lysis were not different between the groups.

**Conclusion:**

In the early stages of sepsis, the development of SAE is correlated with increased systemic clotting activity where platelets seem to have an important role. More research is needed to investigate the role of platelets and the coagulation system in relation to the development of early SAE.

## Introduction

Sepsis is a global health problem, estimated in a 2017 report to affect almost 49 million people and to cause 11 million deaths, representing almost a fifth of all global deaths worldwide [1]. According to Sepsis-3 definition, sepsis is caused by a dysregulated immune response to infection, and diagnosed clinically by the development of new organ dysfunction due to infection [2]. Neurological dysfunction is one of the most common complications seen both in early and late stages of sepsis, affecting from one third to up to 70% of septic patients [3,4,5]. The neurological dysfunction in sepsis is known as sepsis-associated encephalopathy (SAE), a diffuse cerebral impairment caused by the host response to infection in the absence of a direct central nervous system (CNS) infection or other causes for encephalopathy [6, 7]. In a recent study including more than 4000 septic patients from the Medical Information Mart for Intensive Care IV (MIMIC-IV) and the eICU databases in the United States, SAE defined as a Glasgow Coma Scale score less than 15 or abnormal neurological findings consistent with delirium occurred in up to 68% of patients [8].

The spectrum of clinical manifestations of SAE is large, ranging from mild neurological dysfunction manifested as physiological and behavioral modifications to the most severe forms of delirium and coma [7, 9,10,11]. Due to the variable clinical manifestations, the diagnosis of SAE can be challenging especially in the early stages of sepsis, and the incidence, prevalence and mortality associated with SAE are difficult to assess [3, 4].

The pathophysiology and the complex mechanisms involved in SAE are still not completely elucidated. There are several factors involved, such as macro-and microcirculatory disturbances, dysfunction of the blood–brain barrier, neuro-inflammatory processes, or dysregulation of neurotransmitters [4, 12,13,14,15]. Until now, there is not any biomarker validated for the prediction or confirmation of SAE [4, 16, 17]. It is extremely important not to delay the diagnosis of neurological dysfunction, especially in patients with mild symptoms such as somnolence or behavioral changes, which sometimes represent the first clinical signs of organ dysfunction in sepsis. The identification of an organ dysfunction such as SAE leads to an earlier diagnosis of sepsis, allowing the detection of patients with highest morbidity and mortality and a quicker escalation of therapy [4, 18, 19].

It is known that the majority of patients with sepsis present different degrees of coagulation activation, ranging from mild coagulation disturbances to overt disseminated intravascular coagulation (DIC), associated with microcirculatory dysfunction, micro-thrombosis and organ dysfunctions [20, 21]. Cerebral hypoxic/ischemic lesions are commonly found in postmortem studies in patients with sepsis [12, 13, 22, 23]. These can be related to decreased cerebral flow and autoregulation failure, but also to hypercoagulability, microcirculatory dysfunction and endothelial damage [7, 13]. These coagulation changes leading to intravascular thrombosis and sometimes to simultaneous bleeding events are known and commonly seen in severe patients in later stages of sepsis, but the hemostatic changes associated with early development of organ dysfunction in sepsis are much less studied. Therefore, the aim of this study was to assess the coagulation profile in patients with early SAE and to compare the hemostatic parameters between septic patients with and without SAE in the first 24 hours from sepsis diagnosis.

## Methods

### Patient selection

This retrospective observational study included patients with sepsis defined according to the Sepsis-3 criteria [2]. The patients were included in the study in the first 24 hours after sepsis diagnosis. We excluded patients with associated diseases or treatments that lead to coagulation disturbances, such as liver cirrhosis (Child Pugh classes B and C), moderate and severe chronic kidney disease (KIDGO stages 3, 4 and 5), anticoagulant and antiplatelet therapy, pro-coagulant treatments including blood products in the last week prior to enrolment. The use of fractionated or unfractionated heparins in thromboprophylactic dosages was allowed.

The retrospective data was obtained from 2 large-volume centers. The ethical approvals for the study were obtained from the institutional ethics committees of each center (approval numbers 29438/2016, 2257/22.01.2024), which waived the need for informed consent due to the non-interventional nature of the study and the use of data obtained during the routine care of patients. According to institutional regulations, the patients provide the written consent for the use of data obtained during routine care at hospital admission.

### Sepsis-associated encephalopathy (SAE)

The diagnosis of SAE was established based on clinical examination after excluding other diseases, such as cerebrovascular diseases or central nervous system infection, brain injury due to trauma, brain neoplasms, metabolic encephalopathy, and drug side effects. Imaging studies (brain CT) were performed in selected patients based on neurological examination and recommendations to exclude other cerebrovascular diseases, and only patients without other diagnoses explaining their altered neurological were considered to have SAE. The neurologic assessment of the septic patients to identify SAE included the level of consciousness (using Glasgow Coma Scale) and delirium assessment using the Confusion Assessment Method for the Intensive Care Unit (CAM-ICU). Patients were considered to have SAE if they presented altered consciousness and/or delirium. The diagnosis of delirium was based on the presence of at least one positive CAM-ICU screening criterion.

### Blood tests

The blood samples were obtained in all patients in the first 24 hours after study inclusion. In 162 patients, only a complete blood count (CBC) was available. In 118 patients, complete blood count and other hemostatic tests including standard coagulation tests (Prothrombin time, fibrinogen, INR), plasma levels of both pro- and anticoagulant factors, D-dimer levels, and Rotation thromboelastometry (ROTEM, Instrumentation Laboratory, USA) were performed.

Whole blood coagulation was assessed using a ROTEM Delta device with standard reagents and materials from the manufacturer. The technology used was described in detail in other publications [24,25,26]. Standard citrated blood samples were maintained at room temperature and testing was performed within 60 min after blood draw. The ROTEM Delta device used was calibrated weekly as per manufacturer's recommendations. For the current study we used the results of the extrinsically activated assays EXTEM, FIBTEM and APTEM. In EXTEM assay coagulation is activated with tissue factor after blood is recalcified. FIBTEM is modified from EXTEM by the addition of a potent platelet inhibitor (cytochalasin D), leading to the lack of platelet contribution to clotting in this specific test. APTEM is also an extrinsically-activated test that also contains an antifibrinolytic agent, allowing for a better detection of fibrinolytic activity when the two assays (EXTEM and APTEM) are compared [25, 26].

The following parameters were assessed from the EXTEM assay: CT (clotting time, in seconds), CFT (clot formation time, in seconds), MCF (maximum clot firmness, in millimeters), CLI60 (clot lysis index at 60 min after CT), MCE (maximum clot elasticity). The CLI60 represents the remaining clot amplitude at 60 minutes after CT expressed as a percentage of the MCF. The following indices are obtained from the first derivative of the clot firmness curve using the ROTEM Delta device's software: Maximum Velocity (MaxVel), Time to MaxVel of clot formation (t-MaxVel) and area under the curve (AUC). The analysis of the first derivative of the clot formation trace allows to obtain information about the kinetics of thrombus formation reflected by the dynamic clotting parameters. MaxVel is the peak rate of clot formation and t-MaxVel corresponds to the time from the beginning of the reaction until the occurrence of maximum velocity [27,28,29]. The initiation phase of coagulation is reflected by CT and t-MaxVel, the propagation phase by MaxVel and CFT, while the MCF, AUC and MCE are measures of clot strength. Similarly, CLI60 was measured from APTEM, and the difference between CLI60 in APTEM and EXTEM was calculated, in order to assess the decrease in clot firmness after MCF due to lysis. From FIBTEM, MCF and MCE were recorded. Similar to previous publications, we assessed platelet component based on the difference between clot elasticity in EXTEM and FIBTEM (MCE EXTEM-MCE FIBTEM) [30]. This parameter reflects better the platelet contribution to clot strength, which is different from both platelet count and platelet function [30].

### Statistical analysis

Continuous variables were tested for normality using the Shapiro–Wilk test. Data with normal distribution were reported as mean ± SD. If significantly skewed, median and interquartile range (IQR) were used as appropriate. For comparison of continuous variables between 2 groups, the Student's t-test and Mann–Whitney U-test were used as appropriate. Chi-square test was used for comparison of categorical variables. Following univariable analyses, multivariable analysis was performed using variables for which the unadjusted P values were less than 0.1. For this study, a P value of <0.05 was considered significant. Statistical analyses were performed using SPSS Statistics v 23.0 (IBM Corp).

The statistical power of the study was calculated post-hoc using G-Power program aiming for a moderate significant difference in the platelet count between patients with and without early SAE (Cohen's d=0.5) and a type I α error=0.05. The statistical power obtained was 97.17%.

## Results

This retrospective observational study included 280 patients with sepsis (52.1% men), with a mean (±SD) age of 69.64 (±15.27) years. 184 patients (65.7%) presented sepsis-associated encephalopathy (SAE) at study inclusion.

The parameters from the CBC, the age and gender of patients were compared between patients with and without SAE for the whole study group (n=280). The results are presented in Table 1. Following univariate analyses, the variables with unadjusted p values less than 0.1 were used for multivariate logistic regression analysis. Among the variables tested, age, leucocyte and platelet count were selected to be included in the multivariable logistic regression model (Table 2). After adjusting for confounders such as age and leucocyte counts, only the platelet count was useful to predict early SAE.

**Table 1.** Demographic data and complete blood count parameters in patients with and without sepsis-associated encephalopathy (SAE).

**Parameter**	**Patients with SAE (n=184)**	**Patients without SAE (n=96)**	**Reference ranges**	**P value**
Age (years)	73 (18)	70 (16)		0.088
Male sex	94 (51.08%)	52 (54.16%)		0.624
Leucocyte count (per μL)	14100 (10960)	10265 (10882)	4000–9000	0.003^*^
Hemoglobin (g/dL)	10.1 (3.9)	9.8 (3.8)	11.5–17	0.276
Platelet count (per μL)	200000 (166500)	128500 (149500)	150000–400000	<0.001^*^

Data are expressed as median (IQR) or n (%). P value was obtained using the Mann Whitney U test or Chi-square test, as appropriate.

* p statistically significant (<0.05); SAE, sepsis-associated encephalopathy

**Table 2.** Multivariate logistic regression model using age and complete blood count parameters.

**Parameter**	**Adjusted OR**	**95% CI**	**P value**
Leucocyte count	1.000	1.000; 1.000	0.403
Platelet count	1.003	1.001; 1.006	0.004^*^
Age	1.003	0.987; 1.020	0.710

OR, odds ratio; CI, confidence interval;

* P statistically significant.

The hemostatic parameters from SCTs, plasmatic levels of coagulation factors and inhibitors, and ROTEM data were available in 118 patients. For both groups of patients (with and without SAE), the median values of prothrombin time were slightly increased compared to upper normal limit of the laboratory, while the mean plasma levels of both pro and anticoagulant factors were lower compared to the reference ranges provided by the laboratory (Table 3).

**Table 3.** Hemostatic tests in patients with and without sepsis-associated encephalopathy (SAE).

**Parameter**	**Patients with SAE (n=63)**	**Patients without SAE (n=55)**	**Reference ranges**	**P value**
PT (s)	15.95 (3.8)	17 (5.2)	10.4–14.3	0.440
Fibrinogen (mg/dL)	431.88 (±198.14)	373.94 (±170.94)	200–400	0.095
Factor II (%)	57.46 (±15.12)	57.07 (±14.73)	70–120	0.888
Factor V (%)	54.35(±19.13)	52.70 (±18.10)	70–120	0.635
Factor VII (%)	36.33(±13.78)	37.61(±15.54)	55–165	0.638
Factor X (%)	56.34(±16.40)	54.67(±15.62)	70–120	0.579
AT (%)	65.44(±17.85)	68.25(±14.45)	80–120	0.389
PC (%)	58.98(±11.94)	60.06(±13.71)	70–150	0.663
PS (%)	44.86(±10.08)	43.68(±12.04)	60–140	0.581
D-dimer (ng/mL)	4830.5 (6273)	3940 (7493)	<500	0.446
EXTEM CT (s)	69 (20.5)	70.5 (22.8)	38–79	0.791
EXTEM CFT (s)	90.5 (80)	114.5 (104)	34–159	0.001^*^
EXTEM MCF (mm)	64.30(±14.41)	56.71(±12.60)	50–72	0.004^*^
EXTEM MCE	218.81(±126.33)	147.85(±73.31)	-	0.001^*^
EXTEM CLI60 (%)	98(3)	98(4)	>85	0.167
MaxVel (mm/min)	17 (9.8)	14 (10)	11–25^a^	0.021^*^
t-MaxVel (s)	85 (69.5)	99 (105.5)	147–223^a^	0.931
AUC (mm x 100)	6360.16(±1364.86)	5602.31(±1269.07)	-	0.005^*^
FIBTEM MCF (mm)	21.71(±11.40)	16.40(±7.78)	9–25	0.007^*^
FIBTEM MCE	38.44 (±53.24)	20.66 (±13.23)	-	0.024^*^
APTEM CLI60 (%)	98 (3)	98 (4)	-	0.278
Diff CLI60	0 (0)	0 (1)	-	0.374
Platelet component	175.5 (156.75)	119.5 (105)	-	0.010^*^

INR, international normalized ratio; PT, prothrombin time; AT, antithrombin; PC, protein C; PS, protein S; AUC, area under the thrombus formation curve; CFT, clot formation time; CT, clotting time; EXTEM CLI60, lysis index at 60 min; MaxVel, maximum velocity of clot formation; MCF, maximum clot firmness; MCE, maximum clot elasticity; t-MaxVel, time to maximum velocity of clot formation; Diff CLI60, the difference between APTEM CLI60 and EXTEM CLI60; Platelet component, the difference between EXTEM and FIBTEM MCE; SAE, sepsis-associated encephalopathy;

a The reference intervals for MaxVel and t-MaxVel are according to Andersen et al. [18]; Data are expressed as mean (±SD), median (interquartile range, IQR) for quantitative data or n (%) for qualitative data;

* P statistically significant (<0.05).

The data were compared between patients with and without early SAE (Table 3). The results showed a similar coagulation initiation in both groups of patients without any significant differences in PT values, plasmatic levels of pro- and anticoagulant factors, and clotting times.

**Fig. 1.**
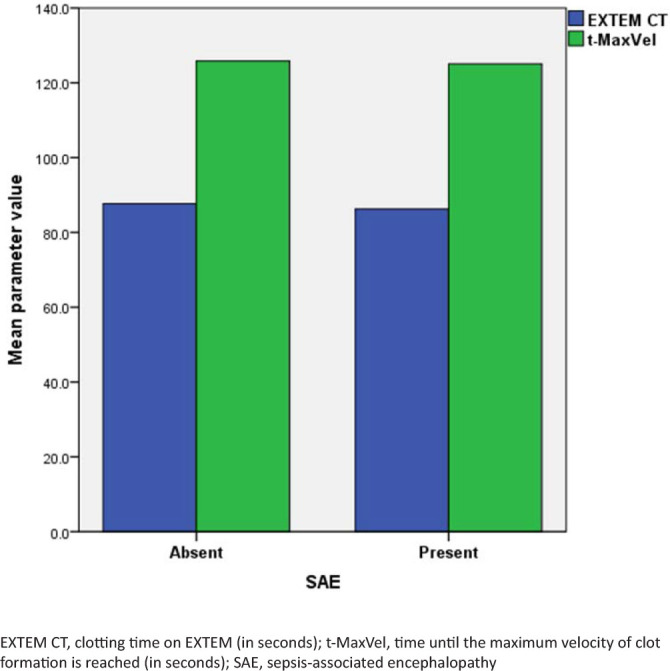
Comparison of coagulation initiation between groups.

**Fig. 2.**
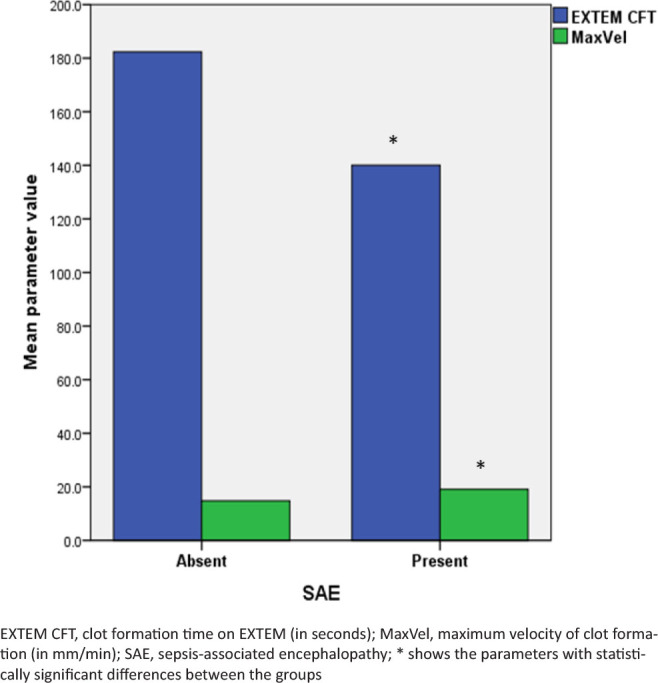
Comparison of coagulation propagation between groups.

**Fig. 3.**
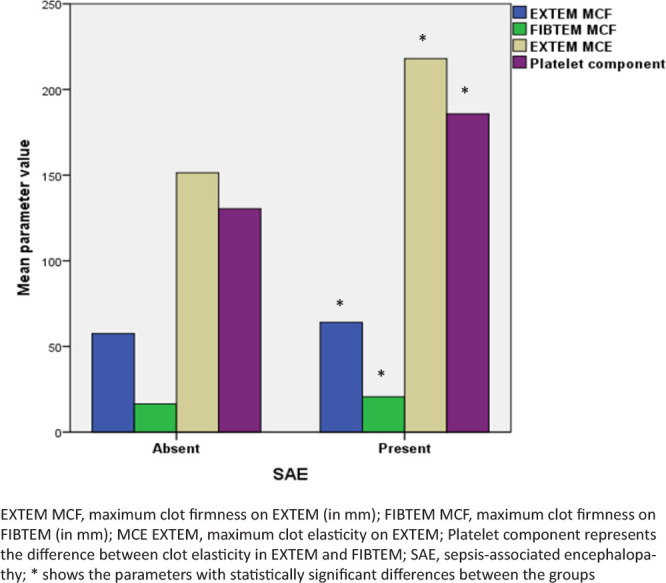
Comparison of clot strength between groups.

The viscoelastic parameters reflecting clotting initiation were not different between the patients with and without SAE (Figure 1). However, the patients with early SAE presented a faster and more active coagulation propagation, with higher velocities of clot formation and shorter clot formation time on ROTEM (Figure 2, Table 3). The clot firmness, elasticity, and platelet component were also higher in patients with SAE compared to patients without SAE (Figure 3, Table 3). Clot lysis was not different between the groups. Although the plasmatic fibrinogen levels were not different between the groups, ROTEM analysis revealed a higher clot strength in FIBTEM in patients with than in patients without early SAE.

To compare the significance of platelet contribution or fibrinogen contribution to the overall increased clot strength that correlates with early SAE, plasmatic fibrinogen and the platelet component were selected to be included in a multivariable logistic regression model. The results show that platelet component remained a good predictor of SAE after adjusting for fibrinogen levels (Table 4).

**Table 4.** Multivariate logistic regression model using fibrinogen levels and platelet contribution to clot strength.

**Parameter**	**Adjusted OR**	**95% CI**	**P value**
Fibrinogen level	1.000	0.997; 1.002	0.403
Platelet component	1.007	1.001; 1.013	0.02^*^

OR, odds ratio; CI, confidence interval;

* P statistically significant.

## Discussion

The main result of this study is that the early development of SAE is correlated with a higher platelet count, with faster coagulation propagation phase, and increased clot strength. Our results suggest that patients with early SAE present with more important platelet contribution to clot formation and strength compared to patients without SAE.

Thrombocytopenia and plasmatic coagulation disturbances are frequent findings in sepsis, ranging from mild abnormalities to overt DIC in severe cases [21]. The severity of hemostatic alterations and of thrombocytopenia is usually correlated with the severity of the disease and the outcome of septic patients. However, this applies to later stages of sepsis, as in the early phases of sepsis the platelet count and coagulation parameters are usually maintained or even in the hypercoagulable range. In severe infections and sepsis, there is a bidirectional interaction between inflammation and coagulation, leading to an activated coagulation system together with dysfunctional anticoagulant and fibrinolytic pathways [31]. This initial coagulation activation is often not detected in clinical practice, but in later phases will lead to the consumption of coagulation factors and platelets, endothelial dysfunction and microvascular thrombosis which contributes to organ dysfunctions in severe cases [31, 32].

Rotational thromboelastometry (ROTEM) is a whole-blood point-of-care test useful for global assessment of hemostasis. It reflects the contribution of both plasmatic and cell-based coagulation during each phase of the coagulation process: initiation, propagation, clot formation and strength, and clot lysis[25]. Previous studies using ROTEM revealed a hypercoagulable profile in patients with early stages of sepsis compared to healthy controls [33]. Both hypercoagulability and hypocoagulability identified by ROTEM in septic patients were correlated with worse outcomes compared to septic patients with normocoagulability [34].

Besides their role in hemostasis, platelets play important roles in inflammation and immune defense, becoming highly activated in sepsis. Activated platelets expose phospholipids on their membranes contributing to the amplification of coagulation, and release mediators which will activate other circulating platelets. The activated platelets can adhere to the endothelium, bind von Willebrand factor and fibrinogen, trigger the neutrophils to release Neutrophil Extracellular Traps, and form platelet-leucocyte aggregates, bringing an important contribution to microcirculatory damage and intravascular clotting in septic patients [35, 36]. The involvement of platelets in different sepsis-associated organ dysfunctions was already described in literature [36,37,38]. Increased platelet activation was documented in ARDS, in sepsis AKI, and in septic cardiomyopathy in human or experimental studies [39,40,41,42,43,44]. In order to address the platelet contribution to organ dysfunction, antiplatelet strategies were proposed based on positive results from animal studies [45, 46]. However, contradictory results were obtained from human studies [47,48,49,50].

To our best knowledge, there is no previous publication assessing the correlation between platelet counts or hemostatic parameters and early sepsis-associated neurological dysfunction. Our study has some limitations. First, this is a retrospective cohort suitable only for further hypothesis generation. Second, the extended panel of hemostatic tests including plasmatic levels of coagulation factors and inhibitors and viscoelastic tests was not available in the whole study group. The size of the study group having complete hemostatic testing was small and a larger number of septic patients would be necessary to confirm our findings. Third, only onetime point testing was performed, not accounting for the dynamic changes in the coagulation state that are possible and important to follow in these complex patients. Another limitation is the lack of other laboratory methods for the specific quantification of important hemostasis components such as thrombin generation, platelet function tests and enzymatic fibrinolysis.

## Conclusion

In early stages of sepsis, a more active coagulation system revealed by faster propagation phase and higher clot firmness and elasticity is correlated with the development of SAE. According to our results, the platelets seem to have an important role, as patients with early SAE presented a higher platelet count, and a higher platelet contribution to clot strength. More research is needed to investigate the role of platelets and the coagulation system in relation with the development of early SAE.
